# Activity of IQG-607, a new orally active compound in a murine model of *Mycobacterium tuberculosis* infection

**DOI:** 10.1186/1753-6561-8-S4-O8

**Published:** 2014-10-01

**Authors:** Diogenes Santos

**Affiliations:** 1 PUCRS, Porto Alegre, Rio Grande do Sul, Brasil

## 

Tuberculosis (TB) caused by Mycobacterium tuberculosis, remains a major global health concern. According to the World Health Organization (WHO), TB was responsible for the occurrence of 1.8 million deaths in 2012 [1], and currently represents the main cause of human death due to a single pathogen. Increasing HIV-TB co-infections, the emergence of multidrug-resistant (MDR) [2,3], extensively drug-resistant(XDR) [3,4], and, more recently, of totally drug resistant strains (TDR) [5] have increased the need for developing new drugs to treat TB. Globally, 3,7% of new TB cases and 20% of previously treated patients are estimated to have MDR-TB [2] Ideally, novel anti-Tb drugs, should be effective against the resistant strains, decreasing the length of treatment thereby improving patients compliance with lower dose frequency, minimal drug-drug interactions and reduced toxicity issues [1,3].

Isoniazid (INH) is the most prescribed drug for active TB and prophylaxis. It has been demonstrated that its primary target is the M. tuberculosis 2-trans-enoyl-ACP (CoA)reductase enzyme (inhA) [4]. Furthermore, INH is s pro-drug, activated by the mycobacterial KatG-encoded catalase-peroxidase enzyme in the presence of manganese ions, NAD(H) and oxygen [5]. Unfortunately, the use of INH has been allied to a series of collateral effects, especially neurotoxic and hepatotoxic side effects [6]. Our group has described a new approach to the rational design of an INH analogue based on an inorganic group (a pentacyanoferrate III/II) attached to the nitrogen atom of the heterocyclic ring of INH, which inhibits the validate target inhA [6,7]. The metal center of the compound can promote an electron transfer reaction that mimics the in vitro activation of INH by KatG enzyme. In fact, our group has also proved that this new compound pentacyano(isoniazid)ferrate(II) (named IQG-607), does not require the activation by katG or any other enzyme to bind to its orimary target the M. tuberculosis inhA [7,8]; this profile might help to overcome an important mechanism of INH-resistance, the missense mutations in the katG gene. Moreover, our group has demonstrated that the compound IQG-607 is able to inhibit in vitro activity of wild-type and INH-resistant I21V,I47T and S94A M. tuberculosis inhA mutant enzymes more slowly than INH [8,10]. Slow rates of dissociation are expected when we intend to reach higher inhibitory effectiveness and, therefore, the intervals between the doses administered to patients can be longer.

Noteworthy, another new compound containing a pentacionaferrate and an oxadiazole moiety, pentacyano(2-metil-5-(piridin-4-il)-1,3,4-oxadiazole)ferrate (II), (named IQG-639) was also found to be able to inhibit the in vitro activity of wild-type and INH resistant (S94A) M. tuberculosis inhA enzymes. Other experiments demonstrated that both IQG-607 and IQG-639 were active against culture of M. tuberculosis H37Rv and two INH-resistant clinical isolates, showing satisfactory efficacy in vitro.

Importantly, we have established toxicological parameters in mice to determine the safety of both compounds and to guide us in the next in vivo tests. Furthermore,90 days repeated-doses toxicological studies in rats revealed a very favorable outcome for IQG-607 which was also very active in mouse macrophages infected with M. tuberculosis. It thus appears warranted to examine the potential in vivo anti-TB activity of these two compounds, IQG-607 and IQG-639. Accordingly, the activity of these compounds was evaluated by using an in vivo murine model of tuberculosis infection. Swiss mice were infected with M. tuberculosis H37Rv strain and IQG-607 and IQG-639 (250mg/kg) were administered during 28 or 56 days. A dose response study was also performed with IQG-607 at 5, 10, 25, 100, 200 and 250mg/kg. The activity of test compound was compared with that of positive control drug INH at 25 mg/kg. After either 28 or 56 days of treatment, both IQG-607 and INH significantly reduced M. tuberculosis-induced splenomegaly, and also significantly diminished the colony forming units (CFU) in both spleens and lungs. IQG-607 or INH ameliorated the lung macroscopic aspect, reducing the lung lesions (granuloma like) to a similar extent [9]. However, IQG-639 was not capable or significantly modifying any evaluated parameters. In addition, experiments using early and late controls of infection revealed a bactericidal activity for IQG-607 in the animal model of infection. The promising activity of IQG-607 in M. tuberculosis-infected mice brings the hope that this compound might represent a good candidate for clinical development as a new antimycobacterial agent. Experiments to demonstrate efficacy and security in dogs and mini-pigs are under way.
